# Childhood TB in HIV ERA - in sub saharan africa

**DOI:** 10.1186/2197-425X-3-S1-A346

**Published:** 2015-10-01

**Authors:** MN Anwar

**Affiliations:** Richmond Chest Hospital, TB & HIV, Richmond, South Africa

## Introduction

TB is surging much of the Africa because of HIV epidemic, it is observed that TB rate is much higher then what is reported it in public health system in developing country. In South Africa out of all TB case 16% are children.

## Objectives

Childhood mortality is increasingly higher in sub-Saharan Africa. To know the reason of child mortality- is it because of TB or HIV or both?

## Methods

TB & HIV infection in children in retrospective study was in our mind. WHO endorsed in 2010 new diagnostic tool to confirm TB, like Gene XPRT, MDR/RIP, PCR based test even in children. Presently it is not widely available in Sub Saharan Africa because of high cost. South Africa lucky to have Gene XPRT test kit available even in district hospital. BCG vaccine and Tuberculin skin test and its results interpretation helps for diagnosis and prognosis of childhood TB. In children, malnutrition, measles, and whooping cough increase the risk of progression to active TB disease.

## Results

Present deaths Worldwide, HIV: 6000/day, TB: 5000/day South Africa,-TB case Incidence: 4th in the world, Childhood TB- 16% of all TB cases. In 2010, 8.8 million new TB cases globally, 1.1 million deaths (excluding HIV).1.1Million new HIV associated TB cases worldwide, Out of all 82% living in Sub Saharan-Africa. About 50-60% of all HIV patients when start with ART, had TB in their life time. Co-infection of TB &HIV - Globally- 13%, South Africa- are around 25- 60% of amongst children. It is observed that the patterns of TB symptoms are also changing to that of conventional one.

## Conclusions

Problem persists still on diagnosis of TB as 87% of the TB patients shows smear negative even with fluorescent microscope. It shows that smear negative TB patients have high mortality even with proper TB treatment. It also observed that HIV patients having low CD4 count had low TB organism in sputum. TB is a multisystem disease. TB is number one cause of death, in HIV infected patients specially children. TB and HIV are correlated with each other; if we can decrease the incidence of HIV we can decrease the incidence of TB both in morbidity and mortality, I will discuss all these issue and facts in this topic.
